# Brown crusts on infant owing to contact dermatitis associated with ethanol disinfection

**DOI:** 10.1002/jgf2.657

**Published:** 2023-11-06

**Authors:** Yuki Matsuhisa, Tsuneaki Kenzaka, Hideo Hirose, Tadao Gotoh

**Affiliations:** ^1^ Department of Pediatrics Center for Community Medicine, North‐Western Gifu Prefecture National Health Insurance Shirotori Hospital Gifu Japan; ^2^ Department of General Medicine Center for Community Medicine, North‐Western Gifu Prefecture National Health Insurance Shirotori Hospital Gifu Japan; ^3^ Department of Internal Medicine Hyogo Prefectural Tamba Medical Center Tamba Japan; ^4^ Division of Community Medicine and Career Development Kobe University Graduate School of Medicine Kobe Japan

**Keywords:** brown crusts, contact dermatitis, ethanol disinfection, infant

## Abstract

A 2‐year‐old girl developed dermatitis with atypical brown crusts after ethanol disinfection. Since many Oriental people have genetically reduced acetaldehyde dehydrogenase type 2 (ALDH2) activity, ethanol disinfection causes acetaldehyde to accumulate in the skin, resulting in dermatitis.

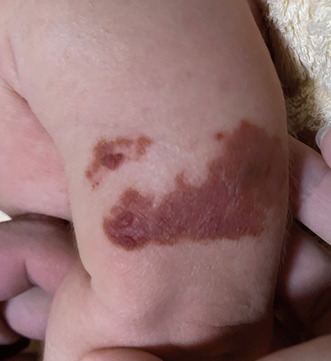

## 1 CASE

**FIGURE 1:**
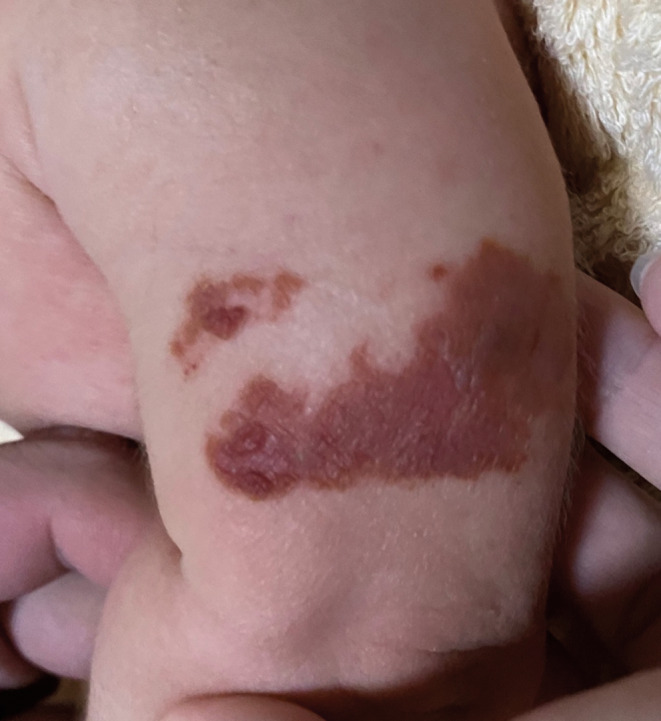
Brown crusts on the left upper arm the day after ethanol disinfection.

A 2‐month‐old girl with no history of illness received her first immunization 2 days prior to her presentation. She received the first oral dose of the rotavirus vaccine (Rotarix®); thereafter, her left upper arm was disinfected with ethanol (83% ethanol + distilled water), and then she received the first subcutaneous injection dose of the hepatitis B vaccine (Beamgen®). Subsequently, the disinfected area on her left upper arm gradually became erythematous and crusted. The next day, brown crusts were observed on the distal dorsal aspect of the left upper arm, around the area where the hepatitis B virus vaccine was administered (Figure 1).

When the second dose of the hepatitis B virus vaccine (Beamgen®) was administered after disinfection with 0.1% chlorhexidine gluconate, no erythema, swelling, or crust formation occurred at the vaccination site. Based on the symptoms and course of the disease, a diagnosis of ethanol disinfection‐associated dermatitis was made.

Many Oriental people, including the Japanese, are genetically predisposed to a decreased activity of aldehyde dehydrogenase type 2, which degrades acetaldehyde, a metabolite of ethanol.^1^ For this reason, the mechanism of alcohol‐induced dermatitis in Oriental people is thought to be primarily a nonimmune reaction in which ethanol that penetrates the skin is decomposed by alcohol dehydrogenase in the skin into acetaldehyde, which accumulates and dilates the surrounding small blood vessels, resulting in redness.^2^, ^3^ Ethanol has also been shown to cause irritation and dryness of the skin.^4^

The patient's erythema soon after disinfection with ethanol was contact urticaria, though the subsequent development of thick brown crusts, which was atypical, has not yet been reported in the literature.

## AUTHOR CONTRIBUTIONS

Yuki Matsuhisa contributed to investigation. Yuki Matsuhisa and Tsuneaki Kenzaka contributed to writing—original draft preparation. Yuki Matsuhisa, Tsuneaki Kenzaka, Hideo Hirose, and Gotoh T contributed to writing—review and editing. All authors have read and agreed to the published version of the article.

## FUNDING INFORMATION

None.

## CONFLICT OF INTEREST STATEMENT

The authors have stated explicitly that there are no conflicts of interest in connection with this article.

## ETHICS APPROVAL STATEMENT

The requirement of ethical approval and consent for this case report were waived because of the retrospective nature of the study by the ethics committee of Center for Community Medicine in North‐Western Gifu Prefecture. This study was carried out in accordance with the guidelines of the Declaration of Helsinki.

## PATIENT CONSENT STATEMENT

Written informed consent was obtained from the patient's parents for the publication of this case report and accompanying images. A copy of the written consent is available for review by the editor of this journal.
